# An ultrasound-based model for predicting the response to neoadjuvant chemotherapy in early stage triple negative breast cancer patients

**DOI:** 10.1186/s12880-025-01818-7

**Published:** 2025-07-08

**Authors:** Yuyang Tong, Yi Wei, Peixuan Sun, Cai Chang

**Affiliations:** 1 Department of Ultrasound, Fudan University Shanghai Cancer Center, Department of Oncology, Shanghai Medical College, Fudan University, 270 Dong’an Road, Shanghai, 200032 China; 2 Department of Ultrasound, Ruijin Hospital, Shanghai Jiaotong University School of Medicine, Shanghai, China; 3 College of Health Science and Technology, Shanghai Jiao Tong University School of Medicine, Shanghai, China; 4 Diagnostic Imaging Center, Shanghai Children’s Medical Center, School of Medicine, Shanghai Jiao Tong University, 1678 Dongfang Road, Shanghai, 200127 China

**Keywords:** Pathological complete response, Neoadjuvant chemotherapy, Triple negative breast cancer, Ultrasound, Nomogram

## Abstract

**Background:**

The accurate identification of patients with triple negative breast cancer (TNBC) likely to achieve pathological complete response (pCR) to neoadjuvant chemotherapy (NAC) holds significant clinical value. The aim of this study was to establish a prediction model that incorporate clinical data and ultrasound features to predict pCR among TNBC patients as early as possible after the initial two NAC cycles.

**Methods:**

From January 2016 to December 2021, a total of 262 patients were recruited and divided into training and validation groups at a 7:3 ratio. Both univariate and multivariate logistic regression analyses were conducted to identify independent factors predicting pCR in the training group. Subsequently, a nomogram integrating the predictive factors was established and applied to the validation group. The performance of this model was assessed based on its discrimination, calibration and clinical utility.

**Results:**

The nomogram that incorporated patient age, clinical T stage, posterior echo enhancement and tumor volume reduction showed robust performance. It achieved an area under curve (AUC) of 0.818, and recorded sensitivity, specificity, and accuracy of 65.2%, 82.5%, and 75.0% respectively in the training group. In the validation group, the model scored an AUC of 0.776, with sensitivity, specificity, and accuracy of 85.7%, 66.7%, and 73.4%, respectively. The decision curve analysis further indicated that the model provided more benefit than standard treat-all or treat-none approaches in predicting pCR.

**Conclusion:**

This prediction model may assist in predicting pCR to NAC among patients with TNBC, enabling an optimal treatment management in clinical practice.

**Trial registration:**

Not applicable.

## Background

Triple negative breast cancer (TNBC) constitutes 15–20% breast cancer cases and is characterized by the absence of estrogen receptor, progesterone receptor, and HER2 expression [1]. This molecular profile renders TNBC unresponsive to endocrine therapy or HER2-targeted treatments, making systemic chemotherapy the backbone of treatment [2]. While chemotherapy remains fundamental, the treatment landscape for TNBC has evolved significantly in recent years [3]. The incorporation of immune checkpoint inhibitors, particularly pembrolizumab in PD-L1-positive cases, has markedly improved outcomes. The landmark KEYNOTE-522 trial demonstrated that adding pembrolizumab to neoadjuvant chemotherapy significantly increased pathological complete response (pCR) rates to 64.8% compared to 51.2% with chemotherapy alone, while also improving event-free survival [4]. Despite these advances, TNBC continues to be the breast cancer subtype with the poorest prognosis, demonstrating the highest rates of recurrence and mortality while exhibiting substantial heterogeneity in treatment response, with pCR rates ranging from 18 to 60% depending on molecular subtypes and therapeutic regimens [5]. This variability underscores the critical need for reliable early predictors of treatment response.

Preoperative neoadjuvant chemotherapy (NAC) serves as the standard treatment for locally advanced TNBC, offering several advantages: tumor downstaging to facilitate breast-conserving surgery, in vivo assessment of treatment sensitivity, and early eradication of micrometastases [6]. Achieving pCR, defined as the absence of invasive residual disease in both breast and lymph nodes (ypT0/Tis ypN0), has been consistently associated with improved long-term outcomes, including overall survival (OS) and disease-free survival (DFS) [7]. However, the inability to reliably predict pCR during early treatment phases remains a major clinical challenge. Accurate early prediction of NAC response would facilitate response-adapted therapeutic strategies: escalating therapy for non-responders to enhance pCR rates, while de-escalating to reduce toxicity for likely responders [8]. Therefore, developing accurate, non-invasive methods to predict pCR during the early stage of NAC has profound clinical implications for optimizing TNBC management.

Breast magnetic resonance imaging (MRI) stands as the most sensitive technique for monitoring treatment response in patients undergoing preoperative NAC [9]. Nonetheless, MRI come with several limitations, including its high cost, time-consuming nature, and being contraindication for claustrophobic patients or those with metallic objects in the body [10]. In contrast, ultrasound (US) is an affordable, convenient and real-time imaging modality widely employed for monitor NAC treatment and characterizing breast lesions [11]. The breast cancer guideline from the China Anti-Cancer Association strongly advocates for regular assessment of treatment response using US after every two cycles of NAC. Currently, utilizing conventional US findings as an early surrogate biomarker for pathological response in patients with TNBC has been rarely reported. Besides, there are no clinically accepted biomarkers are available for early prediction of pCR [12]. Given that pre-treatment US imaging captures the characteristics of the primary tumor and the dynamic changes in tumor volume directly reflect response status [13], we hypothesized that a US-based prediction model incorporating pre- and post-NAC information could accurately predict pCR.

Nomograms offer a graphical representation combining various crucial factors to predict a specific endpoint, potentially aiding clinicians in making individual treatment strategies [14]. In this context, we aimed to establish and validate an easily accessible prediction model that incorporates clinicopathological and US features to predict the pCR among patients with TNBC as early as possible after initial 2 cycles of NAC.

## Materials and methods

### Study cohorts

Due to the retrospective study design, the informed consent was waived by the Institutional Review Board of Fudan University Shanghai Cancer Center, and the study was conducted according to the Declaration of Helsinki. Clinical trial number: not applicable. Patients with core needle biopsy confirmed TNBC, who underwent NAC followed by surgery between January 2016 and December 2021 were enrolled in the study. The exclusion criteria were the following: (i) prior local treatment or systemic chemotherapy; (ii) the lesions were undetectable by US or beyond the measurement scale of US; (iii) incomplete clinicopathologic or US data; (iv) bilateral breast with lesions or ipsilateral breast with multiple lesions; (v) distant metastasis during NAC. Patients with bilateral or multifocal disease or distant metastasis were excluded to ensure standardized measurement of tumor volume changes in a single target lesion, as these conditions may introduce variability in ultrasound assessment and treatment response evaluation.

**Fig. 1:**
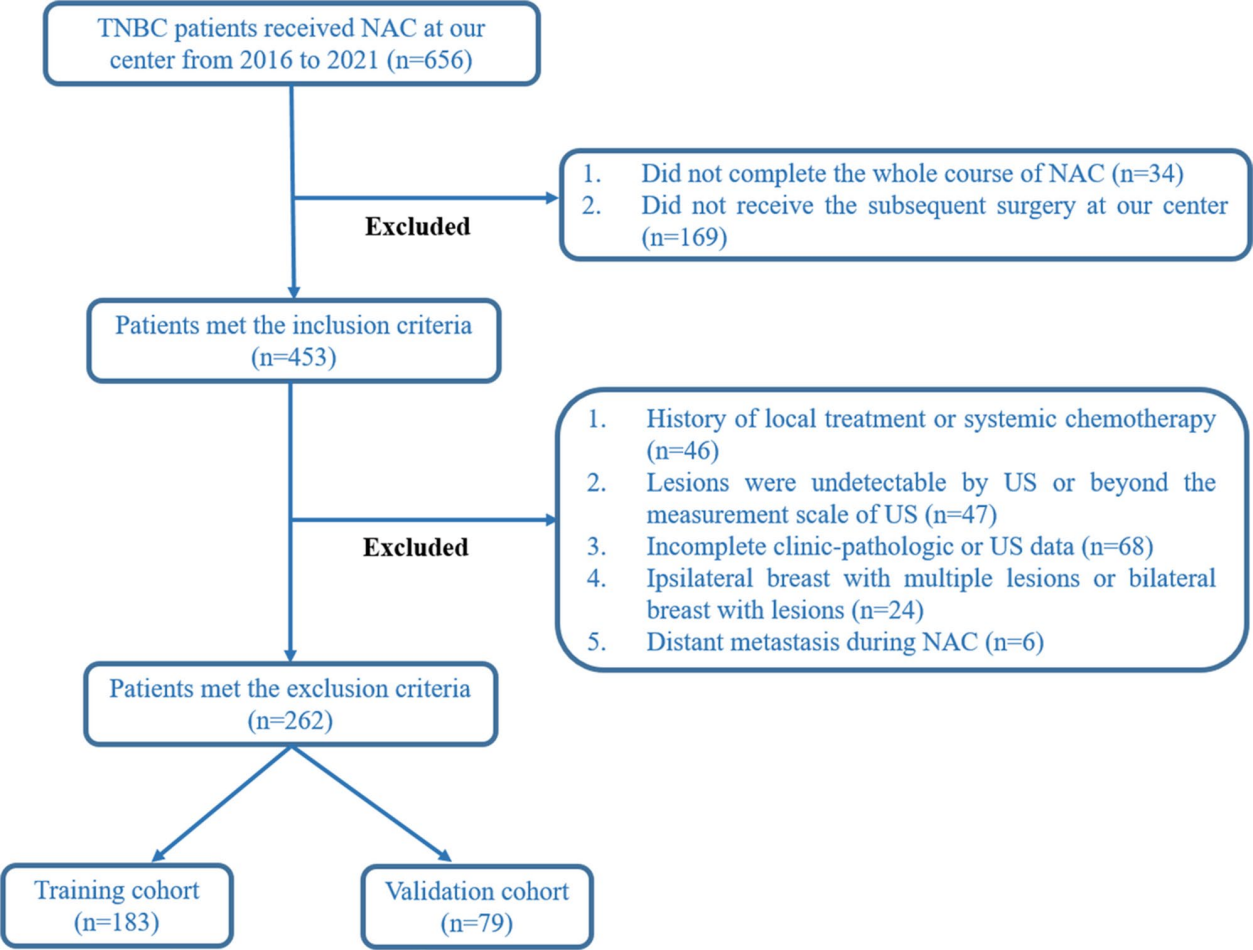
The flowchart illustrates the patient selection process for this study. US, ultrasound; NAC, neoadjuvant chemotherapy; TNBC, triple negative breast cancer

Patients were consecutively and retrospectively enrolled from Fudan University Shanghai Cancer Center between January 2016 and December 2021. All included patients completed both baseline US examination before treatment and follow-up US after 2 cycles of NAC. The cohort was divided chronologically into: (1) a training set of 183 patients treated from January 2016 to December 2019, and (2) an internal validation set of 79 patients treated from January 2020 to December 2021. This time-stratified division was performed without any patient selection or matching procedures, and no statistical adjustments (e.g., propensity score matching) were applied to the baseline characteristics presented in Tables 1 and 2. The detailed patient recruitment process was shown in Fig. 1.

**Table 1:** Patients’ characteristics in the training and validation groups

Characteristics	Training (*n* = 183)	Validation (*n* = 79)	*p*
pCR			0.728
Yes	69 (37.7)	28 (35.4)	
No	114 (62.3)	51 (64.6)	
Age			0.791
≤ 45	82 (44.8)	34 (43.0)	
>45	101 (55.2)	45 (57.0)	
Age			0.402
Mean ± SD	47.3 ± 11.0	48.6 ± 12.5	
Menopausal status			0.481
Pre-menopause	99 (54.1)	39 (49.4)	
Menopause	84 (45.9)	40 (50.6)	
Ki67			1.00
<20%	10 (5.5)	4 (5.1)	
≥ 20%	173 (94.5)	75 (94.9)	
TVR			0.609
Mean ± SD	61.6 ± 27.9	59.7 ± 28.0	
TVR			0.591
<70	93 (50.8)	43 (54.4)	
≥ 70	90 (49.2)	36 (45.6)	
Tumor size (mm)			0.258
Mean ± SD	37.4 ± 16.4	35.1 ± 13.7	
Clinical T stage			0.547
T1	25 (13.7)	10 (12.7)	
T2	126 (68.9)	59 (74.7)	
T3	32 (17.5)	10 (12.7)	
Clinical disease stage			0.519
II	82 (44.8)	32 (40.5)	
III	101 (55.2)	47 (59.5)	
Lymph node metastasis			0.602
Positive	85 (46.4)	38 (50.0)	
Negative	98 (53.6)	38 (50.0)	
Lymphovascular invasion			0.684
Positive	65 (35.5)	26 (32.9)	
Negative	118 (64.5)	53 (67.1)	
Shape			0.328
Regular	26 (14.2)	15 (19.0)	
Irregular	157 (85.8)	64 (81.0)	
Calcification			0.345
Yes	81 (44.3)	30 (38.0)	
No	102 (55.7)	49 (62.0)	
Margin			0.150
Circumscribed	53 (29.0)	30 (38.0)	
Non-circumscribed	130 (71.0)	49 (62.0)	
Orientation			0.173
Parallel	162 (88.5)	65 (82.3)	
Non-parallel	21 (11.5)	14 (17.7)	
Posterior acoustic pattern			0.936
No change	91 (49.7)	41 (51.9)	
Enhancement	32 (17.5)	13 (16.5)	
Shadow	51 (27.9)	20 (25.3)	
Combined pattern	9 (4.9)	5 (6.3)	
Echo pattern			0.249
Hypoechoic	14 (7.7)	11 (13.9)	
Complex cystic and solid	16 (8.7)	5 (6.3)	
Heterogenous	153 (83.6)	63 (79.7)	
US-reported axilla status			0.109
Normal	23 (12.6)	18 (22.8)	
Lowly suspect	24 (13.1)	10 (12.7)	
Highly suspect	136 (74.3)	51 (64.6)	
US-reported supraclavicular status			0.291
Normal	151 (82.5)	71 (89.9)	
Lowly suspect	8 (4.4)	2 (2.5)	
Highly suspect	24 (13.1)	6 (7.6)	

Significant differences are emphasized with bold text

Data are presented as the number of patients and their corresponding percentages unless otherwise noted

SD, standard deviation; pCR, pathological complete response; US, ultrasound; TVR, tumor volume reduction

**Table 2:** Clinical and US characteristics of TNBC patients with NAC grouped by pCR

Characteristics	Training (*n* = 183)			Validation (*n* = 79)		
	pCR	non-pCR	*p*	pCR	non-pCR	*p*
Age			**0.030**			**0.019**
≤ 45	38 (55.1)	44 (38.6)		17 (60.7)	17 (33.3)	
>45	31 (44.9)	70 (61.4)		11 (39.3)	34 (66.7)	
Age			**0.008**			0.191
Median	44.0	50.5		47.5	51.0	
(interquartile range)	38.5–50.5	39.8–58.0		34.3–57.3	39.0–57.0	
Menopausal status			**0.019**			0.934
Pre-menopause	45 (65.2)	54 (47.4)		14 (50.0)	25 (49.0)	
Menopause	24 (34.8)	60 (52.6)		14 (50.0)	26 (51.0)	
Ki67			0.128			0.325
<20%	1 (1.4)	9 (7.9)		0 (0)	4 (7.8)	
≥ 20%	68 (98.6)	105 (92.1)		28 (100)	47 (92.2)	
TVR			**< 0.001**			**< 0.001**
Median	80.4	53.3		81.4	51.6	
(interquartile range)	68.3–92.9	30.9–47.7		72.7–92.1	24.2–73.2	
TVR			**< 0.001**			**0.002**
<70	18 (26.1)	85 (74.6)		6 (21.4)	29 (56.9)	
≥ 70	51 (73.9)	29 (25.4)		22 (78.6)	22 (43.1)	
Tumor size (mm)			0.058			0.091
Mean ± SD	34.5 ± 16.7	39.2 ± 16.1		37.0 ± 13.9	31.5 ± 12.7	
Clinical T stage			**0.013**			**0.008**
T1	16 (23.2)	9 (7.9)		8 (28.6)	2 (3.9)	
T2	43 (62.3)	83 (72.8)		17 (60.7)	42 (82.4)	
T3	10 (14.5)	22 (19.3)		3 (10.7)	7 (13.7)	
Shape			0.601			0.849
Regular	11 (15.9)	15 (13.2)		5 (17.9)	10 (19.6)	
Irregular	58 (84.1)	99 (86.8)		23 (82.1)	41 (80.4)	
Calcification			**0.045**			0.429
Yes	24 (34.8)	57 (50.0)		9 (32.1)	21 (41.2)	
No	45 (65.2)	57 (50.0)		19 (67.9)	30 (58.8)	
Margin			0.177			0.103
Circumscribed	24 (34.8)	29 (25.4)		14 (50.0)	16 (31.4)	
Non-circumscribed	45 (65.2)	85 (74.6)		14 (50.0)	35 (68.6)	
Orientation			0.660			1.00
Parallel	62 (89.9)	100 (87.7)		23 (82.1)	42 (82.4)	
Non-parallel	7 (10.1)	14 (12.3)		5 (17.9)	9 (17.6)	
Posterior acoustic pattern			**< 0.001**			0.154
No change	36 (52.2)	55 (48.2)		14 (50.0)	27 (52.9)	
Enhancement	20 (29.0)	12 (10.5)		8 (28.6)	5 (9.8)	
Shadow	9 (13.0)	42 (36.8)		5 (17.9)	15 (29.4)	
Combined pattern	4 (5.8)	5 (4.4)		1 (3.6)	4 (7.8)	
Echo pattern			0.293			0.972
Hypoechoic	8 (11.6)	6 (5.3)		4 (14.3)	7 (13.7)	
Complex cystic and solid	6 (8.7)	10 (8.8)		2 (7.1)	3 (5.9)	
Heterogenous	55 (79.7)	98 (86.0)		22 (78.6)	41 (80.4)	
US-reported axilla status			0.624			0.939
Normal	7 (10.1)	16 (14.0)		6 (21.4)	12 (23.5)	
Lowly suspect	8 (11.6)	16 (14.0)		4 (14.3)	6 (11.8)	
Highly suspect	54 (78.3)	82 (71.9)		18 (64.3)	33 (64.7)	
US-reported supraclavicular status			0.140			0.406
Normal	60 (87.0)	91 (79.8)		26 (92.9)	45 (88.2)	
Lowly suspect	4 (5.8)	4 (3.5)		0 (0)	2 (3.9)	
Highly suspect	5 (7.2)	19 (16.7)		2 (7.1)	4 (7.8)	

Significant differences are emphasized with bold text

Data are presented as the number of patients and their corresponding percentages unless otherwise noted

NAC, neoadjuvant chemotherapy; SD, standard deviation; TNBC, triple negative breast cancer; US, ultrasound; TVR, tumor volume reduction; pCR, pathological complete response

### Chemotherapy regimens

The study participants received 6 to 8 cycles of NAC following institutional protocols based on National Comprehensive Cancer Network (NCCN) guidelines [15]. The standard regimens consisted of:

Anthracycline + Taxane-based therapy:

Dose-dense AC-T: Doxorubicin (60 mg/m²) + cyclophosphamide (600 mg/m²) every 2 weeks for 4 cycles, followed by paclitaxel (175 mg/m²) every 2 weeks for 4 cycles.

Alternative EC-T: Epirubicin (90 mg/m²) + cyclophosphamide (600 mg/m²) every 3 weeks for 4 cycles, followed by docetaxel (100 mg/m²) every 3 weeks for 4 cycles.

Carboplatin-included regimens: A subset of patients (15.3%) received additional carboplatin (AUC 5–6 every 3 weeks), primarily in clinical trials or high-risk cases.

Treatment intervals and dose adjustments were documented for toxicity management per institutional standards.

### US data acquisition

All patients received baseline US examinations before NAC initiation, followed by serial US assessments after every two cycles, regardless of the variations in chemotherapy regimens or dosing intervals. Whole breast US was performed by six dedicated breast radiologists using high-end equipment including LOGIQ E8 and E9 (GE Healthcare, Wauwatosa, USA), EPIQ 7 and Affiniti 70 (PHIILPS, Bothell, USA) Supersonic Aixplorer System (SuperSonic Imaging, Aix-en-Provence, France) equipped with high-frequency linear-array transducers. All six radiologists involved in the study had at least 5 years of experience in breast US and completed a standardized training session prior to the study. This training included: (1) comprehensive review of Breast Imaging Reporting and Data System (BI-RADS) lexicon criteria; (2) systematic case-based exercises using representative clinical samples to establish interpretation consensus for imaging characteristics. A standardized measurement protocol was implemented for tumor volume assessment: lesions were measured in three orthogonal planes with calipers positioned at the lesion’s outer margins, follow-up measurements were preferentially performed by the initial sonographer, and all measurements underwent verification by a second radiologist. Quality control measures included: (I) periodic review of randomly selected cases by the study coordinator; (II) inter-observer agreement analyses on randomly sampled cases; (III) regular meetings to discuss and resolve any interpretation discrepancies. The baseline US features of the primary breast tumors were assessed according to the BI-RADS lexicon [16], prior to treatment, while tumor volume reduction (TVR) was calculated by comparing baseline and post-cycle 2 measurements. Additionally, regional nodal basins, such as the axillary nodes at levels I to III, and supraclavicular lymph nodes were also evaluated via US. US parameters used for identifying suspicious lymph nodes were as follows: irregular shape, hypoechogenicity, eccentric cortical thickening (> 3 mm), non-circumscribed margin, and the absence of the fatty hilum. Lymph nodes with 3 or more US parameters were considered highly suspicious. By comparison, lymph nodes with less than 3 US parameters were considered of low suspicion. Lymph nodes with none of these criteria were described as normal [17]. All patients with suspicious axillary lymph nodes identified on US, fine-needle aspiration cytology was routinely performed prior to treatment initiation to confirm pathological diagnosis.

The longitudinal and transversal images of the target lesion were acquired, and the measurements of the target lesion in three dimensions were recorded. The volume of the lesion was calculated using the formula:

Volume = 0.52 × Length × Width × Height.

The percentage change in tumor volume between the baseline and after 2 cycles of NAC was determined via the formula:

Chang in Tumor Volume (%) = (V_1_ - V_2_) / V_1_.

where V_1_ represents the tumor volume at the baseline and V_2_ represents the tumor volume after 2 cycles of NAC treatment.

### Histopathology review

Prior to NAC, core needle biopsy specimens of the primary breast cancer were obtained for immunohistochemical staining and evaluation. Immunoreactivity for estrogen receptor (ER) progesterone receptor (PR), HER2, and Ki-67 were reported as the percentage of cells exhibiting positive nuclear staining. TNBC was characterized by < 1% of the invasive tumor cell nuclei showing immunoreactivity for ER and PR [18], and the HER2 negativity was determined in according with the clinical practice guideline of the College of American Pathologists [19].

Surgical specimens were evaluated by pathologists who were dedicated in breast disease. A pCR is defined as the absence of invasive disease in both the primary breast cancer and the regional lymph nodes, with or without residual ductal carcinoma in situ (ypT0/Tis ypN0) [20].

### Statistical analysis

Categorical variables were presented as frequency (percentage), while continuous variables were presented as median (interquartile range). Univariate analyses utilized Student’s t-test, Mann-Whitney U test, and the Pearson’s χ^2^ or Fisher’s exact test, as appropriate. Interobserver agreement for qualitative ultrasound features was quantitatively assessed using Cohen’s kappa (κ) statistics. A random subset of 50 cases was independently reviewed by all radiologists to evaluate consistency in feature interpretation. To assess the clinical relevance of pCR status, we performed retrospective survival analysis on available follow-up data. DFS was defined as time from surgery to first recurrence (local or distant) or death from any cause. OS was defined as time from surgery to death from any cause. Kaplan-Meier curves were generated and compared using log-rank tests. Variables with *p* < 0.05 in univariate analyses were entered into multivariate logistic regression models to identify independent predictors for pCR in TNBC patients. Results are presented as odds ratios (ORs) with 95% confidence intervals (95%CIs). Subsequently, an independent predictive factors-based nomogram model was constructed. Positive predictive value (PPV) curve analyses were conducted to determine the exploratory cut-off point of the percentage reduction in tumor volume between baseline and post-two cycles of NAC that predicted a pCR.

The predictive model’s performance was evaluated using receiver operating characteristics (ROC) curve, calibration curve, and decision curve analysis (DCA). The area under the ROC curve (AUC) was calculated to quantify the of the nomogram’s discrimination efficiency. All statistically analyses were performed with R software (version 3.5.3 http://www.r-project.org) or SPSS version 26.0 software for Mac (IBM corporation, Armonk, USA). The utilized package included “ResourceSelection”, ‘rms’, “caret”, “ggDCA”, “glment”, and “ggplot2”. A significance level of 0.05 (two-sided) was considered indicative of statistical significance.

## Results

### Clinicopathological and US characteristics of the study cohorts

Table 1 summarizes the detailed clinicopathological and ultrasound characteristics of patients from both the training and the validation datasets. Based on the postoperative pathologic findings, the pCR rates were 37.7% in the training dataset and 35.4% in the validation dataset. No significant difference in pCR rate was observed between the two cohorts (*p* = 0.728). Additionally, there were no significant differences observed in any clinicopathological or US features between the two cohorts. Interobserver agreement analysis demonstrated substantial consistency across all evaluated ultrasound features (κ > 0.75 for each characteristic), confirming the reliability of our imaging assessments.

### Survival outcomes by pCR status

**Fig. 2:**
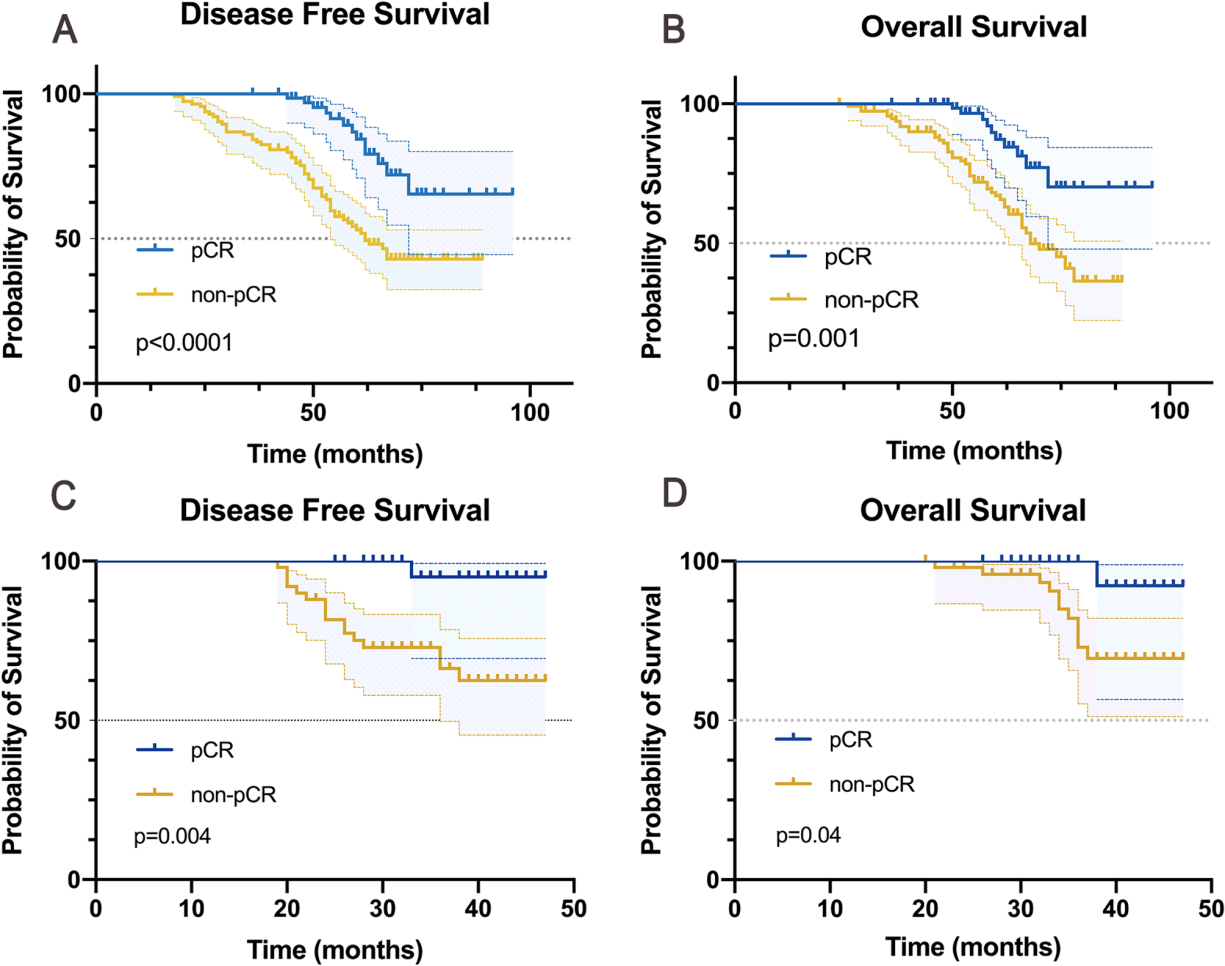
Kaplan-Meier curves of DFS and OS based on pCR status in the training (**A**-**B**) and validation (**C**-**D**) cohorts. (**A**) DFS in the training cohort. (**B**) OS in the training cohort. (**C**) DFS in the validation cohort. (**D**) OS in the validation cohort. Patients achieving pCR (blue lines) demonstrated significantly improved survival outcomes compared to non-pCR patients (yellow lines), underscoring the clinical importance of early pCR prediction. DFS, disease-free survival; OS, overall survival; pCR, pathological complete response

With a median follow-up of 68 months (range 20–96) in the training cohort and 36 months (range27-47) in the validation cohort, pCR demonstrated statistically significant associations with improved survival outcomes in both cohorts. Patients achieving pCR showed markedly better DFS (training cohort: *p* < 0.001; validation cohort: *p* = 0.004) and OS (training cohort: *p* = 0.001; validation cohort: *p* = 0.04) compared to non-pCR patients (Fig. 2).

### Development and validation of the nomogram

Univariate logistic regression analysis revealed a significant association between pCR and the percentage of TVR between baseline and after two cycles of NAC. (*p* < 0.001; OR, 1.044; 95% CI, 1.028–1.061)

**Fig. 3:**
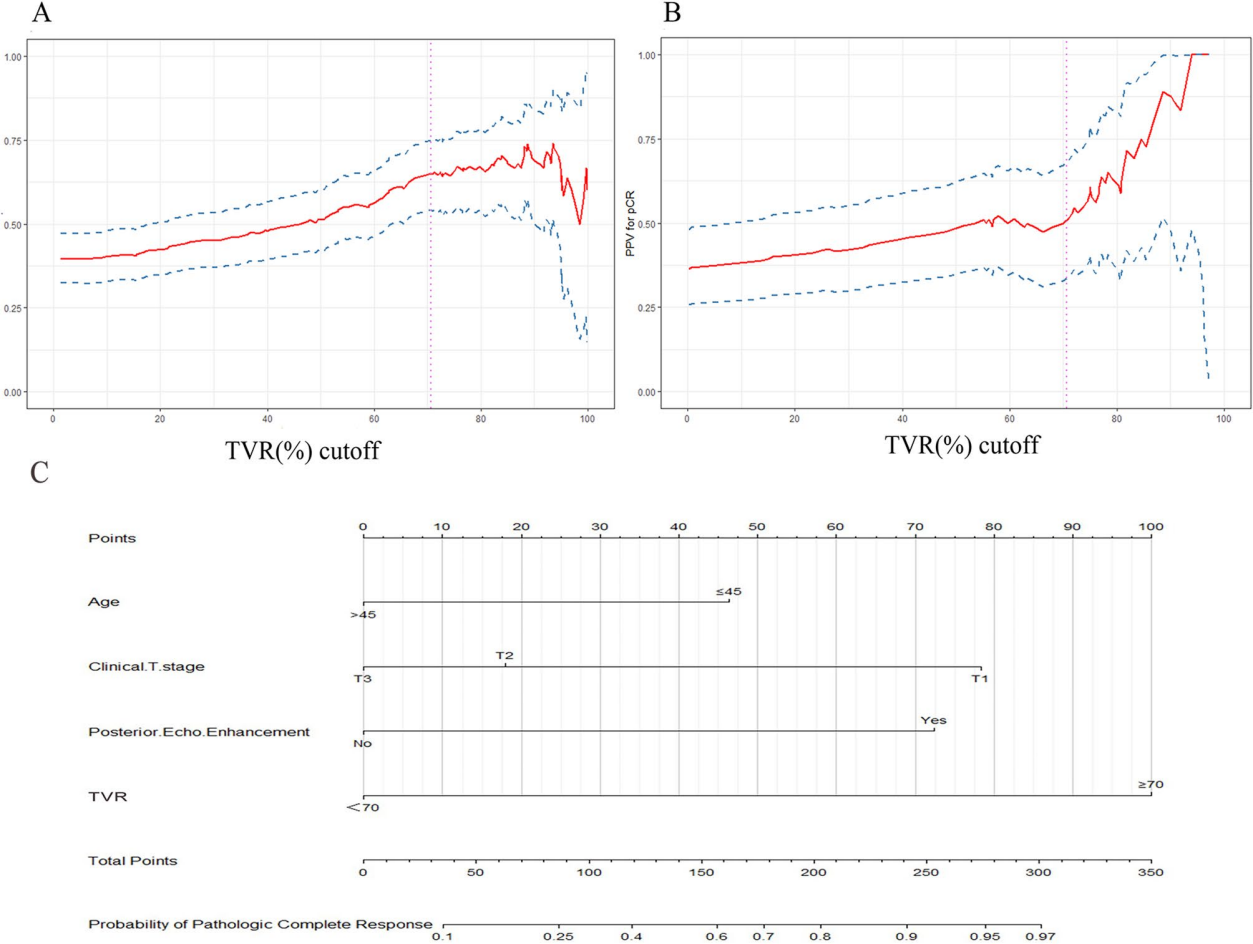
PPV for a pCR by the TVR after 2 cycles of NAC in the training (**A**) and validation (**B**) cohorts. The graphs displayed PPVs of 65.2% and 50.0% for predicting pCR at a 70% volume reduction (dotted vertical lines) in the respective datasets. PPVs were plotted as red solid lines, and their 95% confidence intervals were depicted as blue dashed lines. Nomogram for predicting pCR in patients with TNBC (**C**). The nomogram was developed using the data from the training dataset, incorporating patient age, clinical T stage, posterior echo enhancement and TVR. Each variable’s different values correspond to a point at the top of the graph, where points for all variables are summed and translated into the probability of achieving pCR. PPV, positive predictive value; pCR, pathologic complete response; NAC, neoadjuvant chemotherapy; TVR, tumor volume reduction

A PPV curve analysis indicated an exploratory cutoff point of 70.6% for TVR after two cycles of NAC resulted in the highest PPV of 65.2% (Fig. 3A). To simplify clinical application, we rounded up the cut-off point of 70%. Specifically, in the training cohort, the pCR rate was 63.8% (51/80) and in patients with a TVR ≥ 70% and 17.5% (18/103) in patients with a TVR < 70%. In the validation cohort, the PPV for a TVR ≥ 70% after 2 cycles of NAC was 50% (95%CI, 34.0-68.6%; 22 of 44 patients with a TVR ≥ 70% achieved pCR, Fig. 3B). The pCR rate was 17.1% (6/35) for patients with a TVR < 70%.

**Fig. 4:**
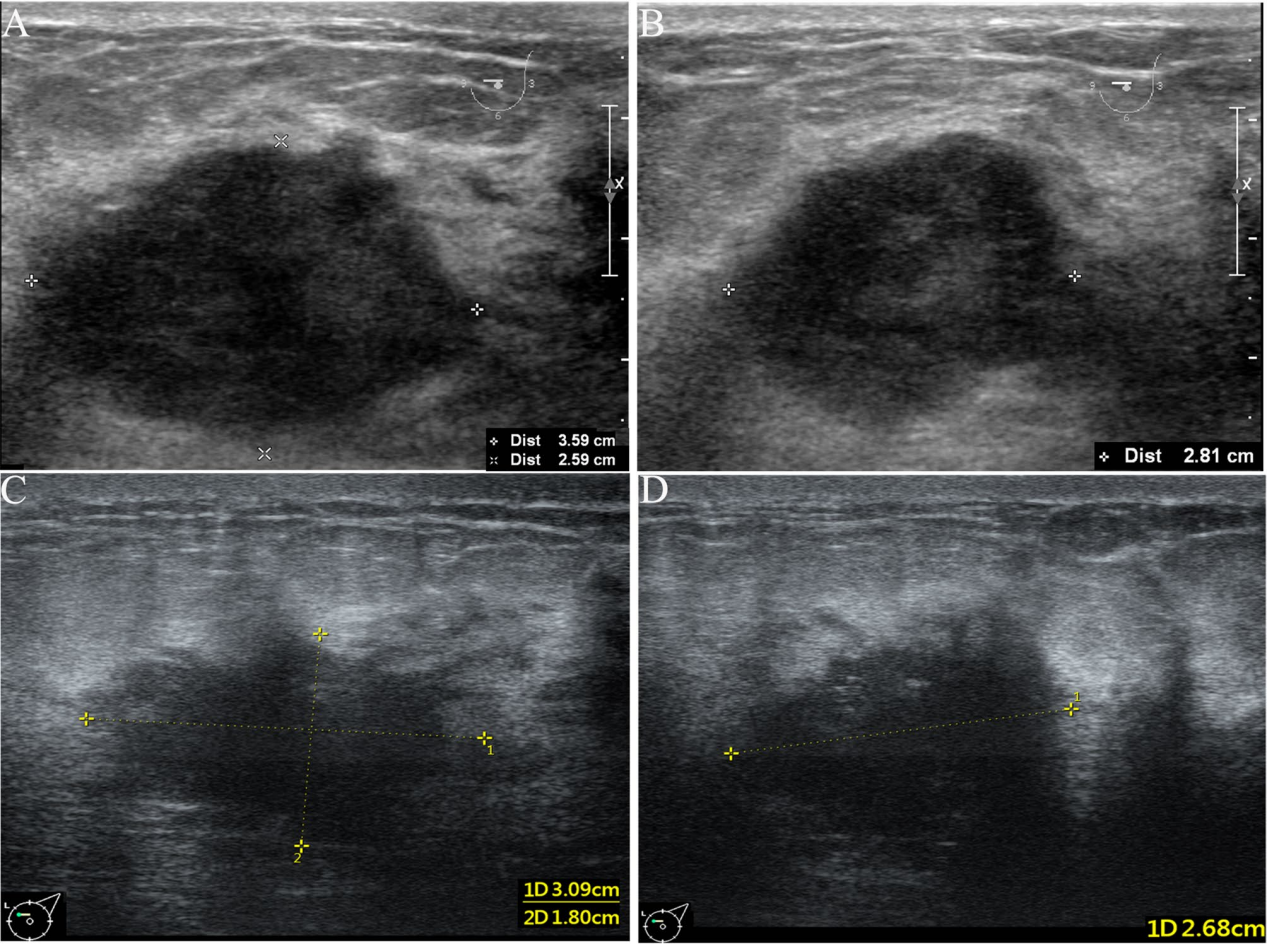
Typical examples of using this nomogram to predict pCR. Images of a female patient (≥ 45 years old, 0 point) with TNBC. (**A**-**B**) Baseline grayscale US images of the primary tumor showed a hypoechoic mass measuring 36*26*28 mm (T2 stage, 18 points) with the unchanged posterior acoustic pattern (0 point). (**C**-**D**) Grayscale US images of the tumor after two NAC cycles showed the tumor measuring 31*18*27 mm with a 43% calculated reduction in tumor volume (0 point). The total points were 18 (0 + 18 + 0 + 0), the corresponding probability of pCR was less than 10%. This patient completed the neoadjuvant systemic therapy, and followed by radical mastectomy, with a surgical pathology evaluated as non-pCR. US, ultrasound; NAC, neoadjuvant chemotherapy; TNBC, triple negative breast cancer; pCR, pathologic complete response

**Fig. 5:**
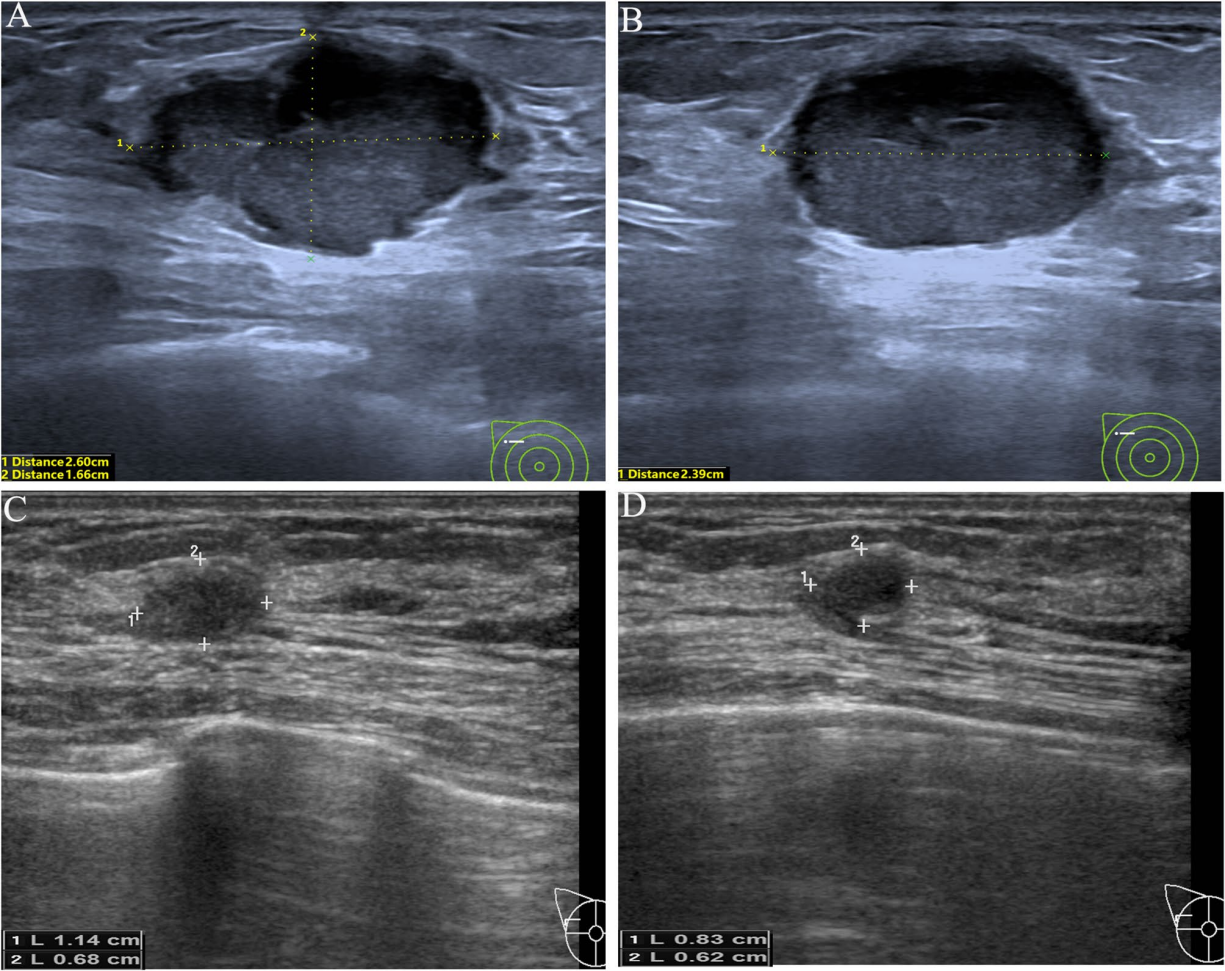
Typical examples of using this nomogram to predict pCR. Images of a female patient (< 45 years old, 47 points) with TNBC. (**A**-**B**) Baseline grayscale US images of the primary tumor showed a hypoechoic mass measuring 26*17*24 mm (T2 stage, 18 points) with the posterior echo enhancement (72 points). (**C**-**D**) Grayscale US images of the tumor after two NAC cycles showed the tumor measuring 11*7*8 mm with a 94% calculated reduction in tumor volume (100 points). The total points were 237 (47 + 18 + 72 + 100), the corresponding probability of pCR was 89%. The patient completed neoadjuvant systemic therapy, and followed by breast-conserving surgery. Surgical pathology evaluation confirmed a pCR. US, ultrasound; NAC, neoadjuvant chemotherapy; TNBC, triple negative breast cancer; pCR, pathologic complete response

Univariate analysis of clinicopathological and US characteristics associated with pCR is presented in Table 2. In the training dataset, significant differences between the two groups were observed in patient age, menopausal status, clinical T stage, calcification, posterior echo enhancement, and TVR. Multivariate logistic regression analysis identified younger age, earlier clinical T stage, posterior echo enhancement and higher TVR as independent predictors for pCR after 2 cycles of NAC (Table 3). These four predictors were then incorporated into the nomogram model (Fig. 3C). Each factor that constituting the nomogram has its corresponding score. The probability of achieving pCR after NAC in patients with TNBC was then generated according to the total points summed up by the score of each factor. Typical cases demonstrating the utilization of the nomogram are illustrated in Figs. 4 and 5.

**Table 3:** Univariate and multivariate logistic regression analyses for predicting a pCR by clinical and sonographic factors

Variable	Univariate			Multivariate		
	β	OR (95% CI)	*p*	β	OR (95% CI)	*p*
Age			**0.031**			**0.009**
≤ 45		1			1	
>45	-0.668	0.513(0.280–0.940)		-0.990	0.372(0.172–0.773)	
Menopausal status						
Pre-menopause		1	**0.020**			
Menopause	-0.734	0.480(0.259–0.890)				
TVR						**< 0.001**
<70			**< 0.001**		1	
≥ 70	1.811	6.118(3.124–11.978)		2.133	8.438(4.012–19.091)	
Clinical T stage			**0.019**			**0.027**
T1		1			1	
T2	-1.233	0.291(0.119–0.714)	**0.007**	-1.287	0.276(0.094–0.759)	**0.015**
T3	-1.364	0.256(0.085–0.774)	**0.016**	-1.671	0.188(0.048–0.677)	**0.013**
Calcification			**0.046**			
Yes		1				
No	0.629	1.875(1.012–3.474)				
Posterior echo enhancement			**0.002**			**0.001**
No		1			1	
Yes	1.244	3.469(1.570–7.665)		1.545	4.687(1.867–12.559)	

Significant differences are highlighted with bold text

OR, odds ratio; TVR, tumor volume reduction; *β*, regression coefficient; pCR, pathologic complete response; CI, confidence interval

### Diagnostic performance of the nomogram model

**Fig. 6:**
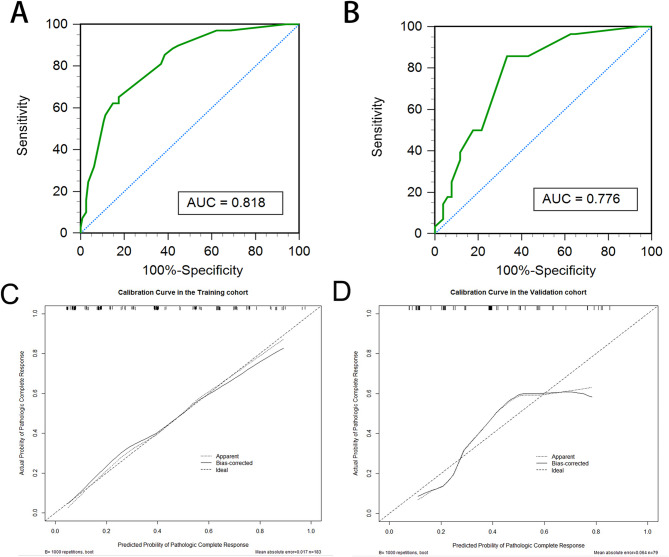
ROC curves of nomogram for predicting the pCR are displayed for the training (**A**) and validation (**B**) cohorts. Calibration curves illustrate the agreement between observed and predicted probabilities for the nomogram in both the training (**C**) and validation (**D**) cohorts. ROC, receiver operating characteristics; AUC, area under the curve; pCR, pathologic complete response

The nomogram model’s diagnostic performance was assessed using the ROC curve, as shown in Figs. 6A-B. In the context of predicting pCR, the model yielded an AUC, sensitivity, specificity, and accuracy of 0.818 (0.756–0.879), 65.2%, 82.5%, and 75.0%, respectively, in the training dataset (Fig. 6A); and 0.776 (0.673–0.878), 85.7%, 66.7%, and 73.4%, respectively, in the validation dataset (Fig. 6B).

The calibration curves in the two datasets indicated a favorable concordance between the pathologic proven ones and nomogram-predicted probabilities (Fig. 6C-D). The Hosmer-Lemeshow test indicated a strong agreement between observations and predictions, with non-significant differences observed (*p* = 0.960 for the training dataset, and *p* = 0.483 for the validation dataset), which suggested that the nomogram has not deviated from an ideal fit.

**Fig. 7:**
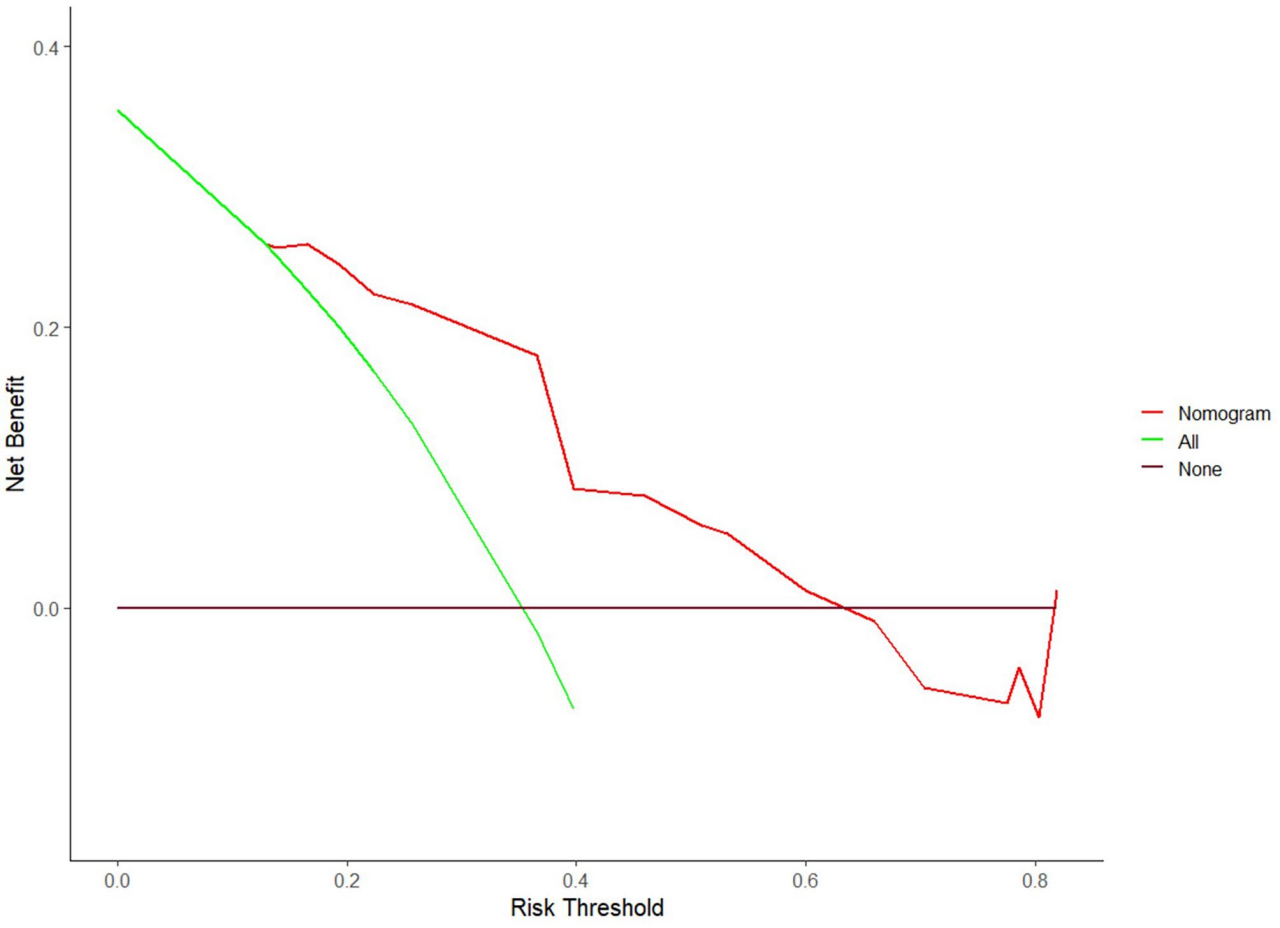
DCA of the nomogram. DCA of the nomogram suggests its utility in predicting pCR in patients with TNBC. Specifically, when a patient’s threshold probability falls within the range of 0.12–0.63, utilizing the nomogram offers a greater benefit compared to treating all or treating none approach of the patients. pCR, pathologic complete response; DCA, decision curve analysis

The DCA based on the prediction model was illustrated in Fig. 7. The DCA revealed that the clinical net benefit of utilizing this model for predicting pCR surpassed that of both the treat-all and treat-none schemes when the threshold probability fell within the range of 0.11 to 0.63.

Taken together, these results demonstrated that our nomogram model had a good performance and clinical value to predict the pCR at an early stage.

## Discussion

Accurate early assessment of treatment response during NAC is clinically crucial for optimizing individualized therapy in breast cancer patients. The I-SPY 2.2 trial has established a response-guided therapy paradigm using early biomarkers to dynamically adjust treatment regimens, significantly improving pCR rates in high-risk breast cancer patients [21]. Aligning with this paradigm, we developed a US-based model for individualized prediction of pCR at an early stage of NAC in patients with TNBC. The prediction model demonstrated favorable performance in discrimination, calibration and clinical utility. This user-friendly graphical tool could facilitate an accurate pCR prediction, potentially assisting clinicians in optimizing the therapeutic strategies to increase pCR rates and improve overall survival.

In this study, the overall pCR rate after NAC was 37.0%, which was consistent with the data in the literature, ranging from 18 to 60% [21]. Our survival analysis confirmed the established prognostic value of pCR in TNBC, with pCR patients showing significantly improved DFS and OS. This finding underscores the clinical importance of early pCR prediction, as patients identified as likely non-responders could potentially benefit from treatment intensification strategies aimed at improving long-term outcomes. The nomogram was developed using the strongest predictive factors, such as patient age, clinical T stage, posterior echo enhancement, and TVR, to predict pCR in patients diagnosed with TNBC. A number of recent studies have highlighted a significant correlation between younger age at diagnosis and achieving pCR [22, 23]. A tumor with an earlier stage indicates a smaller tumor size, and may be easier to be eradicated, we speculate that is the reason why an early T stage is more likely to achieve pCR. Other studies also showed that an early pre-NAC clinical T stage was the significant predictor for pCR [24, 25]. However, several studies demonstrated that T-stage had no statistical correlation with pCR rate [26, 27]. The discrepancy might be attributed to the relatively small sample size of these studies, larger data sets and multicenter studies are warrant to validate the discrepancy.

Tumors characterized by a rapid proliferation rate are more likely to exhibit homogeneous cellularity, leading to reduced attenuation of US wave and increased through-transmission, resulting in posterior echo enhancement [17]. And it is widely acknowledged that fast-growing tumors are more sensitive to chemotherapy. Therefore, the posterior echo enhancement was prone to achieve pCR. Furthermore, TNBC tumors with high stromal tumor-infiltrating lymphocytes (sTILs) were prone to have posterior enhancement, and several studies have shown that higher level of sTILs are correlated with higher rate of pCR in TNBC patients undergoing NAC [28]. These might be the mechanism that lesions with posterior enhancement have a higher pCR rate. The overall decrease in cellularity following NAC is commonly manifested as a reduction in tumor size [29]. Hence, the monitoring of response to NAC via conventional imaging modalities such as US and MRI primarily relies on changes in tumor dimensions. Although MRI-based tumor shrinkage patterns following early NAC served as an additional straightforward indicators of treatment response, the alteration in tumor size remained a robust predictor of pCR in TNBC even after the first cycle of NAC [29]. A recent study showed that a reduction of at least 80% in tumor volume between the initial baseline and after two cycles of NAC on US could effectively predict a pCR in TNBC patients, with an AUC of 0.84 in the primary dataset and an AUC of 0.79 in the validation dataset [30]. Similar to the previous study, a significant TVR on US after 2 cycles of NAC emerged as the most crucial predictor, carrying the maximum weight in the nomogram developed in this study.

The pCR serves as a robust independent prognostic marker of prolonged DFS and OS, and has been widely used as a primary endpoint in clinical trials [31]. Therefore, predicting pCR in the early phase facilitates the decision-making between clinicians and patients. The capacity to preoperatively evaluate pCR using US features, which are less complex, less expensive, and more accessible US features compared to MRI, holds significant potential for both clinical and economic benefits [13]. To our knowledge, this study represents the first attempt to incorporate both clinical and US features, including the US measurements of TVR, to predict the early responses in a relatively large cohort of TNBC patients. All the independent factors comprising the nomogram model in this study were readily accessible, enabling convenient calculation of individual risk scores for pCR as early as the post-cycle 2 US examination, aligning with current personalized care paradigm. Our findings indicated that the US-based prediction model has the potential to guide individualized therapy for patients with TNBC. For patients with no potential for achieving a pCR, the modification of the treatment regimens is necessary to improve the overall pCR rate. In this study, 128 out of 165 (77.6%) non-pCR patients were effectively identified by the prediction model as being likely to benefit from regimens adjustment. In comparison, for those with the potential for achieving pCR, the administration of NAC with the current regimen proves beneficial. Among the 97 patients achieving pCR in this study, accurate predictions enabled 67 individuals to benefit from breast-conserving surgery and the omission of axillary node dissection, which may offer patients an improved cosmetic outcome and better quality of life. For clinical implementation, we propose: (1) standardized US assessment after cycle 2 NAC, (2) nomogram-based pCR probability calculation, and (3) response-adapted therapy strategies. High-probability patients may continue current therapy, whereas low-probability patients should be considered for transition to alternative regimens or clinical trial enrollment. This preliminary study points the direction of our future studies where we need to incorporate more factors and enroll more participants so as to further improve the accuracy to the standard for clinical use.

A number of limitations of this study warrant acknowledgement. Firstly, despite the inclusion of a relatively large dataset, the study remained confined to a single-center study design. Prospective multicenter studies with larger sample size and external validation cohorts are imperative to validate the efficacy of the prediction model before its potential applications in clinical settings. Secondly, while the model exhibited clinically diagnostic capability, its predictive performance, particularly positive predictive value did not reach an optimal level. Further investigations are required to improve its accuracy, potentially through the integration of promising features from state-of-the-art technologies, such as radiomics, contrast-enhanced ultrasound, and elastography. Thirdly, our study excluded patients with multiple lesions, bilateral breast cancer, or distant metastasis during NAC to minimize confounding effects on ultrasound-based measurements. While this approach strengthened internal validity, it may limit the model’s immediate applicability to broader TNBC populations. Future multicenter studies should evaluate the model’s performance in these subgroups to confirm its utility across diverse clinical scenarios. Finally, radiologists performed subjective and qualitative visual assessment of the US features in this study. Further studies should endeavor to integrate artificial intelligence techniques to facilitate reproducible objective and rapid analysis of US images.

## Conclusion

In summary, a prediction model that based on the clinical and US characteristics could serve as a non-invasive biomarker for predicting pCR at an early stage of NAC in patients with TNBC, which might assist clinicians in optimizing chemotherapy regimens and maximizing patient benefit.

## Data Availability

The patient data and ultrasound images supporting this study are not publicly available due to patient confidentiality restrictions under the ethical approval by the Institutional Review Board of Fudan University Shanghai Cancer Center. De-identified data may be made available from the corresponding author upon reasonable request, subject to institutional ethical approval and execution of a data access agreement.

## Abbreviations

pCR: Pathological complete response
NCCN: National Comprehensive Cancer Network
HER2: Human epidermal growth receptor 2
AUC: Area under the ROC curve
BI-RADS: Breast Imaging Reporting and Data System lexicon
TNBC: Triple negative breast cancer
DCA: Decision curve analysis
ER: Estrogen receptor
NAC: Neoadjuvant chemotherapy
CI: Confidence interval
PPV: Positive predictive value
PR: Progesterone receptor
OR: Odds ratio
ROC: Receiver operating characteristics
DFS: Disease-free survival
TVR: Tumor volume reduction
sTILs: Stromal tumor-infiltrating lymphocytes
OS: Overall survival
